# Recent Development of Active Ingredients in Mouthwashes and Toothpastes for Periodontal Diseases

**DOI:** 10.3390/molecules26072001

**Published:** 2021-04-01

**Authors:** Meenakshi Rajendiran, Harsh M Trivedi, Dandan Chen, Praveen Gajendrareddy, Lin Chen

**Affiliations:** 1 The Center for Wound Healing and Tissue Regeneration, Department of Periodontics, College of Dentistry, University of Illinois at Chicago, Chicago, IL 60612, USA; mrajen3@uic.edu; 2 Colgate-Palmolive Company, Piscataway, NJ 08854, USA; harsh_m_trivedi@colpal.com (H.M.T.); dandan_chen@colpal.com (D.C.)

**Keywords:** mouthwashes, toothpastes, active ingredients, periodontal diseases, plaque, gingivitis, periodontitis

## Abstract

Periodontal diseases like gingivitis and periodontitis are primarily caused by dental plaque. Several antiplaque and anti-microbial agents have been successfully incorporated into toothpastes and mouthwashes to control plaque biofilms and to prevent and treat gingivitis and periodontitis. The aim of this article was to review recent developments in the antiplaque, anti-gingivitis, and anti-periodontitis properties of some common compounds in toothpastes and mouthwashes by evaluating basic and clinical studies, especially the ones published in the past five years. The common active ingredients in toothpastes and mouthwashes included in this review are chlorhexidine, cetylpyridinium chloride, sodium fluoride, stannous fluoride, stannous chloride, zinc oxide, zinc chloride, and two herbs—licorice and curcumin. We believe this comprehensive review will provide useful up-to-date information for dental care professionals and the general public regarding the major oral care products on the market that are in daily use.

## 1. Introduction

Periodontal disease is a chronic inflammatory disease of the gingiva and periodontium and is a common cause of tooth loss if left untreated [1,2]. It affects about 20–50% of the population globally and 47.2% of people above 30 years of age in the United States [3,4]. The etiology of periodontal diseases is dental plaque. Dental plaque is composed of microbial biofilms that adhere to the tooth surface [5]. The susceptibility of an individual to develop periodontal diseases depends on the immune and inflammatory responses to the periodontopathogenic bacteria present in the dental plaque and is modified by environmental factors [6,7]. Periodontal diseases are associated with several risk factors such as smoking, stress, poor oral hygiene, and genetic susceptibility [8,9]. Recent studies show that periodontal diseases have close ties with heart and lung diseases as well as diabetes [10,11,12].

Periodontal disease initially begins as gingivitis, which is the most common reversible gum disease characterized by inflammation of marginal and attached gingiva in response to the dental plaque [13]. When dental plaque is left to accumulate, gingivitis may progress to periodontitis, which is the irreversible destruction of the underlying connective tissue and alveolar bone [14,15]. Thus, gingivitis and periodontitis can be prevented and reversed by oral hygiene methods that effectively remove plaque biofilm [16,17]. Treatment for periodontal diseases includes maintenance of daily oral hygiene, supra- and subgingival scaling, root planing, antibiotic medications, and surgical procedures [18,19].

Mechanical plaque control by toothbrushing and flossing are the most recommended and effective methods for maintaining oral hygiene and periodontal health [20]. To supplement the mechanical plaque control, various antimicrobial agents are incorporated in chemical plaque control agents like toothpastes and mouthwashes to inhibit growth of plaque biofilm, particularly in areas of the mouth which are less accessible to toothbrushing [21]. The use of oral care products such as toothpastes and mouthwashes are the key means to prevent periodontal diseases. Chemical plaque control agents have good substantivity in the oral cavity which enables them to maintain oral hygiene between brushings. Various antimicrobial agents or compounds are used in toothpastes and mouth rinses and their efficacy in controlling plaque and preventing gingivitis and periodontitis is well-documented and continues to be studied [22,23,24].

Hence, the aim of this article was to review recent developments in the antiplaque, anti-gingivitis, and anti-periodontitis properties of some common compounds used in toothpastes and mouthwashes by evaluating studies from 2015–2020.

## 2. Chlorhexidine

Chlorhexidine (CHX) is a bisbiguanide with bacteriostatic and bactericidal effects [25]. It is the most studied and most effective anti-plaque and anti-gingivitis agent and is considered the “gold standard” anti-plaque agent [26]. CHX is a broad-spectrum antiseptic agent effective against gram-positive and gram-negative bacteria, yeasts, and viruses [27]. It is a cationic molecule and binds non-specifically to negatively charged membrane phospholipids of bacteria [28]. The mechanism of action of CHX is dose-dependent. It is bacteriostatic at very low concentrations (0.02–0.06%) and bactericidal at higher concentrations (0.12–0.20%) [29]. In addition to its immediate bactericidal effect, CHX also binds to the oral mucosa resulting in a slow and prolonged antibacterial effect [30,31].

CHX is widely used in dentistry. It is available as oral rinses (0.02–0.3%), gels (0.12–1%), sprays (0.12–0.2%), and dental varnishes (1%, 10%, 40%). It is also found in toothpastes and mouthwashes [26,32]. CHX is used most widely as a gluconate compound in disinfectant formulations [33].

The long-term use of CHX is associated with local adverse effects of temporary alteration of taste (dysgeusia) and tooth pigmentation. Unaesthetic brownish pigments accumulate on teeth, tongue, as well as on prosthetic crowns which affects patient compliance [34,35]. It is also shown to have cytotoxic activity against human cells in vitro which can cause apoptosis and necrotic cell death [36].

### Recent Findings

A comparative analysis of three mouthwashes containing CHX showed that a 0.2% CHX mouthwash resulted in a significantly better prevention of supragingival plaque and lower plaque scores than 0.12% and 0.06% CHX mouthwashes after 21 days of use. No significant difference was observed in the plaque inhibitory effects between 0.12% and 0.06% CHX mouthwashes. Furthermore, no differences were seen in gingivitis or the gingival index between the three mouthwashes after three weeks of rinsing [37]. Additionally, the 0.2% CHX mouthwash was observed to significantly reduce plaque, gingival inflammation, and gingival scores when used both with and without alcohol. Rinsing with 10 mL of either solution one time a day for a period of six weeks showed to be more effective in controlling both plaque and gingivitis compared to brushing alone. After six weeks of use, the CHX levels in saliva were higher in both groups using CHX with or without alcohol, with a similar amount of CHX retained in the oral cavity for both groups [38].

The number of studies on CHX has increased over the years and confirms the significant effect of CHX on plaque and dental biofilm. All systematic reviews on CHX confirm it to provide statistically significant improvements of plaque and gingival scores. It has a better anti-dental biofilm and anti-gingivitis properties than the Listerine mouthwash containing essential oils (EO). The relative differences in dental biofilm control were 31.6% and 36% for CHX and 24% and 35% for EO at three months and six months, respectively [39]. Another study showed that a 0.2% CHX mouthwash is more effective than Listerine against the aerobic and facultative bacteria in the supragingival plaque samples from gingivitis patients. CHX produced a zone of inhibition (ZOI) with the average diameter of 18.38 mm after 24 h while Listerine showed no inhibition after 24 h. Furthermore, the mean bacterial count was reduced by 23.13 CFU after using CHX for two weeks while Listerine produced a mean reduction of 19.75 CFU. The antimicrobial effect of CHX persists longer than that of Listerine [40].

Recent studies on CHX have also demonstrated its significant antibacterial effect on periodontal pathogens associated with peri-implantitis. A 0.2% CHX mouth rinse resulted in large zones of growth inhibition on *Aggregatibacter actinomycetemcomitans* species isolated from subgingival plaque samples from peri-implantitis lesions [41]. Another study showed the use of a 0.2% CHX gel during different stages of implant placement to significantly reduce the *Porphyromonas gingivalis* load on the healing abutment. CHX also controls the inflammatory infiltrate in the peri-implant soft tissue which is linked to the bacterial load. Thus, the use of CHX improves clinical outcomes of implant-supported restoration by reducing the presence of *P. gingivalis* and peri-implant inflammation [42]. The use of CHX inside a dental implant abutment connection has also shown to reduce peri-implant marginal bone loss which is caused by bacteria present in the implant connection [43].

A preliminary study of a new oral gel formulation, ADC, named after its active ingredients—Ag+ ions (silver-2-mercaptobenzoate), CHX digluconate, and didecyldimethylammonium chloride, showed its good clinical efficacy when used in daily oral hygiene. The results show a statistically significant reduction in the total bacterial load without any noticeable side effects [44]. The topical application of both the gingiva-adhering and the soluble form of a CHX gel has also shown to improve the clinical parameters after scaling and root planing when compared to no application [45]. Another study showed that when used as an adjunct to non-surgical periodontal treatment in patients with chronic periodontitis, a xanthan-based CHX gel containing 1.5% CHX significantly reduced periodontal pocket depths at selected sites (Mesiodistal: 0.15 mm). The use of CHX alone was not effective due to the high clearance of CHX within the pockets [46].

A recent study assessing the efficacy of a mouthwash containing CHX with fluoride showed that the CHX + Fl combination has a better anti-plaque efficacy than CHX alone. The combination has a better impact on accumulation of plaque while resulting in a drop in plaque pH equal to CHX. These results combined with the previous evidence that a CHX–Fl mouthwash resulted in a better reduction of caries with no reported side effects [47] suggest it to be a promising alternative to CHX mouth rinses [48]. The prominent side effect of teeth staining caused by CHX is also significantly reduced by the addition of an anti-discoloration system (ADS) without affecting the antiplaque activity of CHX [49]. ADS provided significant reduction of stain scores while no differences were seen in the plaque, gingivitis, and bleeding scores [50].

Results of a recent study suggest that CHX in combination with an anti-biofilm peptide 1018 can be used for effective control of oral biofilm growth. The combined use of CHX and a broad-spectrum peptide showed a strong additive effect in killing bacterial cells. Though no difference in residual biofilm volume was seen, the combination resulted in a higher percentage of dead cells compared to the treatment with either CHX or peptide 1018 alone. The proportion of dead cells increased significantly with increasing time of exposure to treatment [51]. According to another recent study, topical CHX, when combined with systemic amoxicillin (AMX) and metronidazole (MET), shows promising results as an alternative approach to intensive mechanical therapy in patients with a severe form of periodontitis. An enhanced non-surgical mechanical therapy combining extensive use of topical CHX with the systemic antibiotics is shown to be effective in the treatment of severe generalized aggressive periodontitis (GAP). The combined therapeutic approaches showed improved clinical parameters and reduced periodontal pathogens. A transitory increase in the minimum inhibitory concentration (MIC) of the subgingival biofilm to CHX and AMX was also seen [52].

In recent years, the use of newer approaches and technology to improve the efficacy of CHX have been studied. Low-intensity direct current (DC) has shown to promote CHX antimicrobial efficacy against *P. gingivalis* within a biofilm. A significant increase in the 0.2% CHX efficacy against *P. gingivalis* was seen when applying 10 mA current. This effect is called a bioelectric phenomenon. No effect of electric current was seen with 1.5 mA [53]. Nanotechnology is also being used to enhance the anti-biofilm efficacy of CHX. A study assessing two forms (spherical and wire) of CHX-encapsulated mesoporous silica nanoparticles (MSNs) show the spherical nanoparticle encapsulated CHX to have a greater antibiofilm capacity than the wire form or the CHX-free form. This is attributed to the effective releasing mode and the close interactions of the spherical form with the microbes [54]. The use of magnetic nanoparticles (MNPs) as a carrier of CHX also shows a great potential in the development of antiseptic nanosystems. CHX attached to MNPs shows an increased ability to inhibit the growth of multispecies biofilms compared to free CHX. The CHX-functionalized nanoparticles did not affect the host cell proliferation or the release of the proinflammatory cytokine, IL8. Findings from the study suggest that MNPs with their unique properties (size, magnetic moment) may be used as a new approach in the treatment of infections caused by drug-resistant pathogenic bacteria [55].

Many recent studies assess the effect of CHX use on the oral microbiome. The use of a CHX mouthwash for seven days exhibited a major shift in salivary microbiome with a significant increase in the abundance of Firmicutes and Proteobacteria species and a reduced amount of Bacteriodetes, phyla SR1 and TM7, and Fusobacteria. This shift was associated with a significant decrease in saliva pH and buffering capacity resulting in more acidic oral conditions favorable for increased dental caries. In addition, the use of CHX reduced the amount of oral nitrate-reducing bacteria which contribute to cardiovascular health. These findings suggest a more careful consideration of the applications of the CHX mouthwash. [56]. In another study, the impact of short-term exposure of CHX on two types of oral biofilms, a human tongue microbiota and a 14-species community, was explored. Both biofilms treated with CHX showed a pattern of inactivation (>3 log units) and rapid regrowth to the initial bacterial concentrations. Profound shifts in microbiota composition and metabolic activity were also seen. The study suggests the need for alternative treatments that selectively target the disease-associated bacteria in the biofilm without affecting the commensal bacteria [57]. A comparative study on the recovery of multispecies oral biofilms following treatment with chlorhexidine gluconate (CHX) and CHX with surface modifiers (CHX-Plus) showed CHX-Plus to be more effective in killing bacteria in biofilms than the regular 2% CHX. Though the cells continued to be killed for up to a week after treatment with both CHX solutions, the biofilms returned fully to the pre-treatment levels after eight weeks. The results indicate the presence of persister cells as the main reason for relapse. These persister cells remain in the dormant state and promote tolerance to high concentrations of CHX. Thus, the study highlights the need to identify compounds that synergize with CHX to prevent regrowth of bacteria while limiting its negative side effects [58]. According to a recent review on evidence for resistance in oral bacteria to CHX, though it is not fully clear if there is a reason for concern regarding enhanced tolerance or resistance in oral bacteria towards CHX, the dental community must be aware about the potential risk of CHX resistance [59].

## 3. Cetylpyridinium Chloride

Cetylpyridinium chloride (CPC) is a cationic quaternary ammonium compound with a broad-spectrum antimicrobial activity [60]. It has been employed as an antiseptic agent in oral hygiene products for the past 50 years [61]. It is both bactericidal and bacteriostatic and rapidly kills gram-positive bacteria and yeast in particular [62,63]. It also has substantial antiviral effects against certain viruses [64,65]. The antiplaque effect of CPC is attributed to the cationic component binding readily to the negatively charged bacterial surfaces and intraoral tissue proteins [66,67,68]. It inhibits cell growth and causes cell death by interacting with the cell membrane and causing leakage of cellular components [69].

CPC mouth rinses produce a small but significant adjuvant reduction of plaque and gingival inflammation when combined with toothbrushing [62]. Although CPC is less effective than chlorhexidine (CHX) in reducing plaque and gingival inflammation [68], it provides an additive effect when combined with CHX, increasing the CHX antimicrobial activity and reducing its adverse effects [70]. CPC has a long history of safe and effective use in oral care [62,68] with limited adverse effects which include gingival irritation and mild teeth staining [71]. The use of CPC mouthwash is also known to be safe and effective during pregnancy. It reduces the severity of periodontal diseases and the incidence of pre- term birth in pregnant women [72].

### Recent Findings

When used as the only oral hygiene product, CPC mouth rinses showed significant reduction of gingival inflammation by disturbing the maturation of dental plaque. CPC oral rinses maintain the composition of healthy plaque in its immature state by preventing the acquisition and accumulation of new bacterial taxa. They thus prevent gingivitis which is caused by mature plaque. They also significantly inhibit 17 gingivitis-associated bacterial genera and reduce the interactions and connectivity of certain gingivitis-enriched taxa [73]. The conclusions of another study assessing the in vitro effects of eight commercial mouthwashes on the virulence factors of *Candida albicans* and a group of viridans streptococci suggest that both CPC- and CHX-containing mouthwashes may provide a positive balance for oral health and maintain the microbial homeostasis of the oral cavity. The results showed that only CPC-containing mouthwashes impaired the adhesion of the yeast form of *C. albicans* to both biotic and abiotic surfaces. Furthermore, CPC-containing mouthwashes impaired the biofilm formation by *Streptococcus salivarius* more effectively than CHX-containing mouthwashes and with fewer side effects thus showing potential as a good alternative to CHX [74].

A comparative study of two new mouthwash formulations containing CHX and CPC (1) 0.12% CHX and 0.05% CPC, 2) 0.03% CHX and 0.05% CPC) showed that 0.12% CHX and 0.05% CPC results in greater levels of plaque reduction after scaling and root planing in patients with chronic periodontitis. The new formulation was more effective than the commercially available mouthwash with the same active ingredients (0.12% CHX and 0.05% CPC) with additional effect on periodontal pathogens [75]. Results from another study showed that a 0.12% CHX and 0.05% CPC mouthwash had the same antiplaque and anti-gingivitis effect as a 0.20% CHX mouthwash. The combination also showed fewer side effects than CHX alone. Addition of CPC enables the percentage of CHX in the mouthwash formulation to be reduced while maintaining the same efficacy of CHX with reduced adverse effects [76]. In addition, a mouth rinse containing 0.03% CHX and 0.05% CPC provided benefits in the management of peri-implant mucositis when used in conjunction with mechanical plaque removal. The oral rinse combination resulted in complete resolution of mucositis in 58% of the study subjects and prevented the development of mucositis in all the subjects [77].

Other new formulations containing CPC have also been developed and tested recently. A novel mouth rinse combining CPC and hyaluronic acid (HA), which is a natural compound with anti-inflammatory and bacteriostatic properties and prevents growth of plaque, showed similar effects to CHX in preventing plaque accumulation and no difference in preventing gingivitis. Furthermore, no adverse effects of CHX were seen. This mouth rinse combination with the synergistic effects of its active molecules is suggested to be a promising alternative for regular home mouthwashes [78]. In a different study, the use of a mouthwash containing CPC and tranexamic acid (TXA), a synthetic derivative of the amino acid lysine with antifibrinolytic activity, was tested in patients with gingivitis. It showed a statistically significant reduction of supragingival dental plaque and alleviated the symptoms of gingival bleeding, particularly, of bleeding on probing (BOP) over a period of six weeks [79]. A chewing gum formulation containing CPC with an analog of decapeptide KSL (KSL-W) which is a broad-spectrum antimicrobial peptide, exhibits great potential as an alternative means of maintaining oral hygiene in those unable to perform routine toothbrushing. The combination showed strong reduction of biofilm viability in a dose-dependent manner along with sustained release of both the antiplaque and anti-microbial agents within 30 min after chewing the gum [80].

In a recent study on pregnant women, the use of a CPC mouthwash not only prevented the progression of periodontal diseases, but also improved the periodontal condition during pregnancy. Furthermore, the CPC mouthwash significantly reduced the risk of premature rupture of membranes which is usually caused by intrauterine infection and host inflammatory response in pregnant women [81].

## 4. Fluorides

Fluorides are primarily used to reduce the prevalence of caries and to enhance enamel remineralization [82,83]. The antibacterial and cariostatic effects of fluorides have been extensively accepted [84] and the widespread use of fluorides has been attributed to the decline of dental caries in Western countries in recent years [85]. Fluorides act primarily by formation of fluorohydroxyapatite crystals which have a greater resistance to organic acids than hydroxyapatite crystals of tooth enamel [86]. It has also shown to reduce organic acid production in cariogenic bacteria such as *Streptococcus mutans* [87].

There are various forms of fluorides available for topical application like toothpastes, gels, foams, varnishes, and mouth rinses [88]. Fluorides are considered to be the most important active ingredient in a toothpaste [89] and fluoride toothpastes are the most widely used method for maintaining a constantly low level of fluorides in the oral cavity [90]. Although fluorides are important for caries prevention, chronic daily ingestion of fluorides greater than 1 mg/l or 0.1 mg/kg during the period of tooth development leads to dental fluorosis which is characterized by hypomineralized enamel formation [91,92]. Clinically, dental/enamel fluorosis appears as mild opaque white or brown mottling of enamel associated with pits and enamel fractures in both deciduous and permanent teeth [93].

Various fluoride compounds have been used over the years, like stannous fluoride (SnF_2_), sodium fluoride (NaF), sodium monofluorophosphate (SMFP), acidulated phosphate fluoride (APF), and amine fluoride (AmF) [94]. The first clinically proven anti-caries toothpaste contained stannous fluoride and was introduced in 1950 [95]. SnF_2_ is still considered to be superior to other fluoride compounds. It is a broad-spectrum antimicrobial agent with effects on dental plaque and gingivitis [96]. The stannous ion (Sn^2+^) is the active form in SnF_2_ and has been shown to exert the antiplaque effect by inhibiting bacterial metabolism and reducing the bacterial virulence/ biomass [96,97]. Stannous ions (Sn^2+^) have a better plaque-inhibiting effect than stannic ions (Sn^4+^) and are hence important to maintain the Sn^2+^ state in the toothpaste/mouth rinse formulation for maximum efficacy [98]. Sodium fluoride is the simplest fluoride compound and is found in most toothpastes. It has a bacteriostatic effect [99]. Free F ions from dissociated NaF pass through the cell membranes of bacteria and interfere with bacterial metabolism [100].

The generally accepted fluoride concentration in toothpastes is 1000–1500 ppm. Higher levels of fluorides (1000 ppm or more) are associated with an increased risk of dental fluorosis in children under 5–6 years of age [101,102]. To prevent the risk of fluorosis in children, only a “smear” layer of low-fluoride toothpastes (500 ppm) on the brush is recommended along with parental supervision [103]. Fluoride mouth rinses typically contain 100–500 ppm fluorides and are recommended for high caries risk individuals such as patients with hyposalivation or patients undergoing orthodontic treatment. The use of fluoride mouth rinses is generally not recommended for children under 6 or 7 years of age who may not know to spit it out completely [89].

### 4.1. Recent Findings

#### 4.1.1. Sodium Fluoride

Use of NaF toothpastes when compared to AmF toothpastes showed to significantly reduce the number of bacteria in all biofilms including the tongue, palatal and buccal mucosa biofilms (where the highest numbers of bacteria were found), and the mouth floor biofilm (which had the lowest number of bacteria). Application of AmF, however, reduced the number of bacteria only in the buccal mucosa biofilm. Following the decrease, the number of living bacteria reached their baseline values in 120 min after the application, thus demonstrating a short-term effect of NaF [104]. The significant decrease in bacterial viability seen on different oral surfaces is suggested to be associated with the immediate increase in fluoride bioavailability which has been demonstrated in previous studies [105,106].

A recent study showed a moderate concentration of NaF to stimulate proliferation and mineralization in periodontal ligament cells (PDLCs) in vitro. NaF showed a dose-dependent effect on cell proliferation when different concentrations were applied to PDLCs cultured in an osteogenic medium. Cell viability was seen to increase from the 50 µmol/L concentration of NaF reaching a peak at 500 µmol/L and declining at a higher concentration of 5000 µmol/L NaF. The cells treated with 10 and 500 µmol/L showed a significant increase in alkaline phosphatase (ALP) activity which indicates an increased potential for bone formation. The results suggest that addition of a suitable concentration of NaF into periodontitis treatment medications like perio packets and tissue patches may provide a novel therapeutic approach for periodontal regeneration [107].

The first published trial evaluating the efficacy of a mouth rinse combining 0.06% CHX with 0.05% NaF showed significant improvement in oral hygiene both clinically and microbiologically. The combined mouth rinse formulation was tested comparatively with 0.06% CHX and 0.05% NaF alone mouth rinses in orthodontic patients who are more susceptible to develop dental caries and gingivitis. It was seen that the use of CHX + NaF induced a significant improvement in the bleeding index (BI), modified gingival index (MGI), and plaque index (PI). These changes in clinical parameters caused by CHX + NaF were found to be slightly but not significantly better than the ones caused by CHX alone. Furthermore, the anticariogenic effect of NaF alone was clearly seen with significant decrease in cariogenic bacteria—*Streptococcus mutans* and lactobacilli. Though the combined CHX + NaF group did not have more effects on *S. mutans*, it showed a synergistic effect and significantly reduced the count of lactobacilli which showed partial resistance to CHX alone [108]. Another study compared the effects of NaF and CHX on the architecture of plaque biofilm formed on enamel surfaces in vivo. Both NaF and CHX produced a similar reduction in overall vitality. It was seen that while CHX exerted its effect particularly on the bacterial cell surface throughout the biofilm, NaF demonstrated deeper levels of biofilm penetration by producing more dead/damaged bacteria in the middle to lower levels of the biofilm [109].

Results from that study also support the combined use of NaF and cetylpyridinium chloride (CPC) in mouth rinses to inactivate oral bacteria and to protect tooth enamel. Addition of NaF did not affect the antibacterial and anti-biofilm efficacy of CPC-containing formulations. Formulations containing 0.075% CPC alone or in combination with 225 ppm NaF equivalently inactivated bacteria in planktonic, ex vivo, and biofilm models and produced a significant viability loss. These data together with the previous findings that CPC does not interfere with the effects of fluoride [110] support the combined use of NaF and CPC in mouth rinse formulations [111].

#### 4.1.2. Stannous Fluoride

Adding an SnF_2_ toothpaste to the daily oral care regimen has been shown to have multiple oral health benefits including reduction of dental calculus buildup, dental plaque, gingivitis, staining, and halitosis. A recent systematic review found evidence that significant reduction in calculus formation was produced by a toothpaste containing SnF_2_ and a calcium phosphate mineralization inhibitor (SHMP) [112]. The SnF_2_ + SHMP toothpaste also showed to play an important role in stain reduction [113,114]. The review also demonstrated a high plaque-inhibitory effect of an SnF_2_ dentifrice. Both short-term and long-term antiplaque effects were seen with the use of SnF_2_, with reductions in dental plaque ranging from 1.6% to 25.8% [115,116,117]. Furthermore, SnF_2_ was found to have both direct and indirect effects on the development of gingivitis. The direct effect referred to an anti-inflammatory action while the indirect effect—to the amelioration of gingivitis caused by plaque reduction [118]. Furthermore, brushing with a toothpaste containing SnF_2_ was more effective than a regular sodium fluoride dentifrice in inhibiting overnight dental plaque regrowth. The effect of SnF_2_ was seen to extend over a 24-h period [119].

A dentifrice containing 0.454% SnF_2_ is shown to have superior efficacy in controlling gingivitis and supragingival plaque over a 24-week period compared to a standard dentifrice. Twice daily brushing with a SnF_2_ dentifrice resulted in a statistically significant reduction in whole mouth gingival bleeding. Significant differences were seen in the bleeding index at 12 weeks and in the plaque index, modified gingival index, and the number of bleeding sites at 24 weeks. With over two thirds of the subjects achieving 10% or fewer bleeding sites, clinically relevant improvement in gingivitis was seen with the use of a 0.454% SnF_2_ dentifrice [120]. A meta-analysis of 18 randomized clinical trials showed further evidence that bioavailable gluconate-chelated 0.454% SnF_2_ dentifrices effectively reduce gingivitis as measured by gingival bleeding. The use of an SnF_2_ dentifrice for ≤ three months reduced the number of bleeding sites equating to a benefit of 59%, 35%, and 67% in mild, moderate, and severe cases of gingivitis, respectively. Thus, large reductions of gingival bleeding were seen with SnF_2_ dentifrices like that observed with flossing. Reduction in gingivitis was seen in all types of gingivitis including generalized gingivitis, localized gingivitis, and isolated sites of gingival inflammation in periodontally healthy cases. Based on these results, using an SnF_2_ dentifrice produced a 3.7 times better chance of transitioning from gingivitis to healthy gingiva [121].

A toothpaste (Colgate Total^SF^) containing 0.454% SnF_2_ stabilized with 1% zinc phosphate has been shown to significantly reduce plaque, gingivitis, dentin hypersensitivity, and extrinsic stains in both in vitro and clinical studies. It effectively controlled plaque biofilm and reduced gingival inflammation over a six-month period when compared with the control fluoride toothpaste. Results from in vitro studies showed that it provided an effective occlusion of dentinal tubules with a deposit consisting of tin, zinc, silicon, and a phosphate. Clinically statistically significant improvement in tactile dentinal hypersensitivity scores and air blast hypersensitivity scores were seen at week 4 and 8 examinations when compared to the control toothpaste. It also demonstrated enhanced extrinsic teeth staining prevention and removal with no adverse effects when compared to a nonabrasive SnF_2_ gel and a regular fluoride toothpaste [122,123,124,125].

SnF_2_ has also been shown to reduce the viability of the biofilm-adhering organisms on orthodontic retention wires in vivo. Major shifts in biofilm composition were seen when combining an SnF_2_ toothpaste with a mouth rinse containing an essential oil. The shift is attributed partially to small changes produced in the bacterial cell surface hydrophobicity after the adsorption of toothpaste components which stimulate bacterial adhesion to the hydrophobic oil in the mouth rinse. The use of an SnF_2_ toothpaste both alone and in combination with the mouth rinse produced a decrease in the prevalence of lactobacilli, *Streptococcus oralis*/*Streptococcus mitis*, and *Streptococcus sanguinis* [126]. For a systematic review on the efficacy of stabilized stannous fluoride (SnF_2_), please refer to a recent publication [127].

## 5. Stannous Chloride

Stannous chloride (SnCl_2_) is another stannous-containing salt which is added to toothpastes and mouth rinses for its significant effect against enamel and dentine erosion. Its anti-erosive effects have been proven in vitro [128,129], in situ [130], and in vivo [131]. It is also an antiseptic agent and its effect against oral bacteria was first detected in 1884 by Miller [132].

### Recent Findings

In a clinical study, the efficacy of pure experimental fluoride solutions (NaF, SnF_2_, AmF, Na_2_PO_3_F) and stannous chloride (SnCl_2_) were evaluated in terms of initial oral bio-adhesion under in situ conditions. While no significant effect was seen with NaF and Na_2_PO_3_ (sodium fluorophosphate), rinsing with both pure SnF_2_ and SnCl_2_ solutions significantly reduced the initial bacterial colonization. No differences were seen between the effects of SnF_2_ and SnCl_2_. An altered pellicle ultrastructure with a thicker basal layer and a thicker and less electron dense granular and globular layer was detected following use of both Sn solutions attributing the effects mainly to the stannous ions’ content. The increase of pellicle thickness reduced the bacterial adhesion (antiadhesion effect) and the bacterial viability. Thus, the protective properties of the pellicle are shown to be improved by both stannous-containing solutions—SnF_2_ and SnCl_2_ [133].

A new experimental toothpaste containing stannous chloride and amine fluoride was developed and its effects on plaque and gingivitis inhibition was examined in comparison with a sodium monofluorophosphate toothpaste. Twelve-week use of the new toothpaste statistically significantly reduced the plaque when compared to the monofluorophosphate toothpaste. While bleeding and gingivitis were also reduced significantly, no clinically relevant differences were observed between the two groups. Among the adverse effects, tooth and tongue discolorations were frequently seen in patients of the test group which were removed completely by dental prophylaxis at the end of the study [134].

## 6. Zinc

Zinc is an essential trace element [135] and is found throughout the body in muscle tissue, bone and skin [136]. It is also present naturally in saliva [137,138,139], teeth [140,141], and dental plaque [142]. Oral mucosa is suggested to be the most important oral reservoir of zinc.

Zinc is added to toothpastes and mouth rinses as an antibacterial agent to control plaque, to reduce oral malodor by inhibition of volatile sulfur compounds, and to reduce calculus formation through crystal growth modification/inhibition. It has a broad-spectrum antibacterial activity [143] and acts mainly by targeting the cytoplasm and glycolytic enzymes of bacterial cells and by inhibiting the process of glycolysis [144]. The inhibitory activity exhibited by zinc salts on microbial glycolysis is dependent on salivary pH and is bacteria-specific. The inhibition is the highest at pH 7 as seen with *Streptococcus salivarius* and *Streptococcus sobrinus* [145]. It has good oral substantivity [146] and is retained in saliva and plaque for many hours following application. In vitro studies show that zinc is taken up by the salivary pellicle by binding to the pellicle-coated tooth surface and desorbing subsequently into the saliva [146,147]. Repeated application of zinc has been shown to produce a buildup effect in plaque [148,149]. Application of zinc has also been shown to prevent dentin demineralization and to promote remineralization [150]. It also reduces enamel and hydroxyapatite (HA) solubility through adsorption onto HA crystal surfaces [151,152,153] and/or incorporation into the crystal lattice [154,155].

Zinc is used in toothpastes and mouth rinses in the form of a variety of salts like zinc oxide, zinc citrate, zinc chloride, zinc lactate, and zinc sulphate [156]. Usually, zinc oxide, zinc citrate, or zinc chloride is used in toothpastes together with fluorides or triclosan. Zinc is also used in certain restorative materials and denture adhesives [157]. Zinc salts also prevent the production of volatile sulfur compounds (VSC) and thus reduce oral malodor [145]. The delivery of zinc depends on the type of zinc salt used and the dosage of the toothpaste or mouth rinse used [158].

According to the Scientific Committee on Consumer Safety, the use of water-soluble zinc salts in toothpastes is considered safe in adults and children aged 0.5–17 years. Since the use of mouthwashes is not recommended for children under 5 years of age, the use of zinc mouthwashes is recommended from 6 years of age. While excessive intake of zinc is relatively rare, some neurological effects like numbness or tingling sensations and nerve damage have been associated with excessive intake of zinc in patients routinely using denture adhesives containing large quantities of zinc [157].

### Recent Findings

The effects of two new fluoride toothpastes containing 0.96% zinc (zinc oxide, zinc citrate), 1.5% L-arginine, and either 1450 ppm or 1000 ppm fluorides such as sodium fluoride in reducing oral bacteria in teeth, multiple soft tissue locations, and in saliva were compared to a toothpaste containing only fluorides. Both new toothpastes with Dual-Zinc plus Arginine resulted in statistically significant reductions of oral bacteria on the teeth, tongue, cheeks, gums, as well as in saliva 12 h after 29 days of twice daily brushing compared to the fluoride only toothpaste. The two test toothpastes were shown to be clinically equivalent and the results demonstrated that regular twice daily use of these new toothpastes provides 12-h antibacterial protection for whole mouth [159].

An experimental dentifrice was synthesized by incorporating zinc oxide (ZnO) nanoparticles and fluoride-doped bioactive glass nanoparticles (F-nBG) and was evaluated for its antimicrobial efficacy and fluoride release behavior. The ZnO–F-nBG dentifrice provided simultaneous benefit in treating early white spot lesions, reducing bacterial growth, and in plaque control. The dentifrice had an effective antibacterial potential against two cariogenic bacteria, *Streptococcus mutans* and *Lactobacillus casei*, with zones of inhibition (ZOIs) and minimum bactericidal concentration being directly proportional to the F-nBG filler loading. The fluoride release was also higher in the experimental dentifrice compared to the commercial dentifrice and was dependent on the filler loading. Furthermore, fluoride release was found to be more efficient from suspensions than from elutes of the samples of the dentifrice. This is suggested to be of great clinical significance since dentifrices are used in the suspension form during brushing [160].

ZnCl_2_ and CPC have been shown to inhibit the growth of seven bacterial strains, *Streptococcus mutans*, *Streptococcus aureus*, *Porphyromonas gingivalis*, *Prevotella intermedia*, *Fusobacterium nucleatum*, *Treponema denticola* and *Tannerella forsythia*, which are known to cause peri-implant disease and halitosis. A commercial mouth rinse containing ZnCl_2_, CPC, or a combination of both was added to the media in the concentration of 0.25% ZnCl_2_ with 2.5% CPC or of 2.5% ZnCl_2_ with 0.25% CPC. Both ZnCl_2_ and CPC suppressed the growth of almost all the tested bacterial strains except *T. denticola*. It was seen that ZnCl_2_ combined with CPC had the greatest inhibitory activity on the growth of almost all the tested strains except for *T. denticola* and *P. gingivalis* which was followed by ZnCl_2_ alone and then CPC, suggesting a possible synergistic effect of the two ingredients. ZnCl_2_ in general was more effective than CPC in suppressing the bacterial growth. ZnCl_2_ showed the most significant inhibitory effect on *P. gingivalis*. Furthermore, while CPC did not have any inhibitory effect on the growth of *T. denticola*, ZnCl_2_ featured some inhibitory effect on it [161].

## 7. Herbs

In recent years, there has been a rise in the use of herbs and plant extracts in toothpastes and mouthwashes. The emergence of multidrug resistant pathogens and the need for economical, safe, and effective alternatives has led to the increase in the use of natural phytochemicals derived from plants in oral hygiene products. Aloe, curcumin, eucalyptus oil, licorice, neem, and tea tree oil are some of the herbs and plant products commonly studied for use in toothpastes and mouthwashes. We focused on licorice and curcumin, two natural products being studied extensively for use in dentistry and oral care products.

### 7.1. Licorice

Licorice, the root of *Glycyrrhiza glabra*, is a herb native to Asian and Mediterranean countries. Due to its sweet taste, licorice is used as a natural sweetener and flavoring agent in foods, drinks, and candies. In addition, licorice roots have been used for centuries in traditional Chinese medicines and Ayurveda due to its numerous health benefits [162,163,164].

The pharmacological effects of licorice are attributed to its rich secondary metabolites like glycyrrhizic acid and glycyrrhetic/glycyrrhetinic acid (GA) [165]. Licorice has many pharmacological benefits, including antimicrobial, antiviral [166], antiulcer [167], anti- inflammatory [168,169], hepatoprotective [168], and immunoregulatory effects [169]. Several studies highlight the benefits of licorice in treatment of various diseases like atherosclerosis, gastric ulcers, tuberculosis, immunodeficiency, hepatic and bacterial infections, and cancer [170,171,172].

Licorice also features potential beneficial effects in oral diseases like dental caries, gingivitis, periodontitis, candidiasis, and recurrent aphthous ulcers. Studies show licorice extracts and bioactive licorice ingredients to have effects on oral microbial pathogens and the host immune response involved in oral–dental diseases [162]. Licorice lollipops were found to be safe and effective against the cariogenic bacteria *S. mutans*, leading to a sharp decline in the salivary bacteria number when used twice daily for a period of 10 days and three weeks [173,174]. The licorice root polysaccharide extract shows strong antiadhesive effects against *P. gingivalis* which is associated with the onset and progression of gingivitis and periodontitis [175]. The study also showed licochalcone A isolated from licorice to inhibit *P. gingivalis* biofilm formation and the host immune response [176]. The licorice extract exhibited potent anti-inflammatory effects against *A. actinomycetemcomitans* and *P. gingivalis* LPS-induced IL-1β, IL-6, IL-8, and TNF-α responses of macrophages [177]. An in vivo study also showed licorice to inhibit the production of matrix metalloproteinases by host cells and to be as effective as doxycycline in patients with chronic periodontitis [178].

Licorice is available in the form of candies, lollipops, capsules, tablets, and liquid extracts. It is listed as “generally recognized as safe” by the FDA in the USA. It is considered safe for individuals who are not sensitive to glycyrrhizin and when consumed in small quantities. According to the WHO, 100 mg/day of licorice can be consumed safely without any side effects [162,170]. However, studies show that continuous exposure to high doses of licorice, particularly to glycyrrhizin, can lead to hypokalemia, severe hypertension, metabolic alkalosis, and edemas due to its hypermineralocorticoid-like effect [170,179].

#### Recent Findings

A recent study in rats showed GA to inhibit periodontal destruction. Topical application of GA, a component of licorice, in the rat gingival sulcus inhibited attachment loss, alveolar bone resorption, invasion of LPS, formation of immune complexes, and infiltration of inflammatory cells in LPS-induced experimental periodontitis [180]. For a comprehensive review of clinical studies summarizing the biological mechanisms of licorice, its therapeutic effects, and potential adverse risks as determined from human clinical trial data in oral diseases, please refer to these two publications [181,182].

### 7.2. Curcumin

Curcumin is a natural polyphenol derived from the plant *Curcuma longa* Linn, commonly known as turmeric. Though turmeric is cultivated principally in India, China, and other Asian countries, it is also common in other parts of the world [183]. It is used as a spice in cooking, as a food colorant, and in cosmetics [184]. It has also been traditionally used in the Chinese medicine and Ayurvedic medicine for thousands of years to treat bacterial infections and inflammatory diseases [185,186].

Curcumin is the primary curcuminoid and the most researched active component of turmeric. It has various therapeutic effects including anti-inflammatory [187,188,189], hypoglycemic [190,191,192], hepatoprotective [193], cardiovascular [189,194,195], antiarthritic [196], and antimicrobial and anticarcinogenic properties [193,197,198]. It has been shown to be potentially beneficial in treating various diseases like diabetes, pulmonary disease, cardiovascular disease, arthritis, Alzheimer’s disease, Parkinson’s disease, wound healing, skin diseases, gastric ulcers, and cancer [199,200,201].

Due to its diversified properties, curcumin also features therapeutic effects in the treatment of oral diseases like gingivitis, periodontitis, and oral cancer [202,203]. Studies show curcumin mouthwashes to have comparable antiplaque and anti-gingivitis effect to CHX mouthwashes [204,205,206]. Massaging with ground curcumin has been shown to eliminate dental pain and swelling and applying a paste of curcumin, salt, and mustard oil provides relief from gingivitis and periodontitis [203]. A local drug delivery system using 2% whole curcumin gel has been shown to be effective in treating chronic localized or generalized periodontitis when used in conjunction with scaling and root planing [207]. Curcumin also potentiates the effects of chemotherapy and radiotherapy and arrests carcinomatous cells [208].

Various formulations of curcumin have been used in studies, e.g., emulsions, liposomal encapsulation, nanoparticles, tablets, capsules, gels, powders, pastes, and mouthwashes [201]. The use of curcumin is considered generally safe with no associated acute toxicity due to its poor bioavailability [209]. Clinical studies show curcumin to be well-tolerated even at doses of 12 g/day [210,211]. However, adverse effects like allergic contact dermatitis [212,213] and contact urticaria [214] have been reported.

#### Recent Findings

There are two recent clinical studies evaluating the comparative efficacy of topical application of a turmeric gel and a CHX gel. In one study, a comparative reduction of plaque and gingivitis was seen with both a turmeric gel and a CHX gel. The turmeric gel showed a better acceptance due to its pleasant odor and absence of teeth staining compared to the CHX gel which was reported to have a bitter taste and teeth staining [215]. The other study showed both a turmeric gel and a CHX gel to be effective in preventing plaque and gingivitis. However, the CHX gel featured better antiplaque effects than the turmeric gel and further studies are recommended to evaluate the substantivity and anti-inflammatory properties of turmeric [216]. Based on the results of these two recent studies and thre other clinical studies where turmeric mouthwashes showed comparative antiplaque and anti-gingivitis effects to CHX mouthwashes [204,205,206], CHX and turmeric can be used as adjuncts to mechanical means of treating gingivitis. However, there is a need for more long-term studies to further evaluate the comparison of turmeric and CHX. For a comprehensive review of clinical studies on the efficacy of turmeric in the prevention and treatment of gingivitis as compared to CHX, please refer to [217].

The use of a 0.1% curcumin mouthwash (prepared by dissolving the curcumin extract in distilled water) in chronic gingivitis patients was found to be comparable to the effect of a 0.2% CHX mouthwash in plaque reduction and better than that of a CHX mouthwash in reducing gingival inflammation and in the reduction of reactive oxygen metabolites. While a non-significant difference was seen in plaque index scores between curcumin and CHX groups, a significant reduction in the gingival index was seen in the curcumin group compared to the CHX group. The results of the clinical study thus suggest curcumin mouthwashes as a potential alternative to CHX mouthwashes because of its anti-inflammatory and antioxidative properties [218].

Another clinical study also showed a 0.1% curcumin mouthwash to have comparable antiplaque and anti-gingivitis efficacy to a 0.2% CHX mouthwash. Curcumin, CHX, and placebo mouthwashes were used by subjects with moderate to severe gingivitis for 28 days. Plaque, gingival, and sulcus bleeding indices were recorded at days 0, 7, 14, and 28. It was seen that the CHX and curcumin mouthwashes had a similar mean relative reduction of the plaque, gingival, and sulcus bleeding indices. The results suggest that both CHX and curcumin mouthwashes can be used effectively as an adjunct to scaling and root planing [219].

A recent study in rats provided new evidence about the inhibitory effect of curcumin on inflammatory activity. Curcumin inhibited IL-1β and TNF-α in LPS-stimulated rat gingival fibroblasts. It inhibited activation of NF-KB and the LPS-induced decrease in the osteoprotegerin (OPG)/soluble receptor activator of nuclear factor kappa B ligand (sRANK) ratio which is a crucial determinant of alveolar bone absorption and metabolism, thus regulating the inflammatory activity in rat periodontitis. It significantly reduced gingival inflammation, collagen fiber destruction and alveolar bone loss in vivo [220].

## 8. Summary

This comprehensive review summarized the current state of evidence primarily based on recent studies from 2015–2020 with respect to the efficacy of various ingredients of toothpastes and mouthwashes in preventing and managing plaque, gingivitis, and periodontitis (Table 1). It can be noted that CHX is the most studied chemical agent followed by CPC and fluorides. Significance of improvements in the clinical parameters is seen in many of the recent studies on the various active ingredients included in this review. Herbs such as licorice and curcumin show potential efficacy like the non-herbal ingredients. However, further studies are required to recommend them as alternatives to the non-herbal ingredients. This review does not conclude which is the most effective agent against plaque and periodontal diseases. However, evidence shows that the use of chemical plaque control agents in toothpastes and mouthwashes is effective in preventing accumulation of plaque and in the prevention and treatment of periodontal diseases.

## Figures and Tables

**Table 1:** Active ingredients in mouthwashes and toothpastes for periodontal diseases.

Active Ingredient	Available Forms	Antiplaque Effect	Anti-gingivitis Effect	Anti-periodontitis Effect	Antimicrobial Effect	Effect on Bacteria	Side Effects
**Chlorhexidine**	Toothpastes outhwashes Oral rinses Gels Sprays Varnishes	√	√	√	Effective against gram-positive and gram-negative bacteria, yeast, and viruses like *Porphyromonas gingivalis, Aggregatebacter actinomycetemcomitans*, Bacteriodetes, Fusobacteria, phyla SR1, TM7	Bacteriostatic and bactericidal	Temporary alteration of taste, pigmentation of teeth, tongue and prosthetic crowns
**Cetylpyridinium chloride**	Mouthwashes Toothpastes Chewing gums	√	√	√	Effective against gram-positive bacteria, yeast, and viruses like *Streptococcus salivarius*, viridans streptococci, *Candida albicans*	Bacteriostatic and bactericidal	Gingival irritation, mild teeth staining
**Fluorides**	Toothpastes Mouthwashes Gels Foams Varnishes	√	√	√	Effective against *Streptococcus mutans*, lactobacilli, *Streptococcus oralis, Streptococcus mitis, Streptococcus sanguinis*	Bacteriostatic and bactericidal	Dental/enamel fluorosis
**Stannous chloride**	Toothpastes Mouthwashes	√	√	×	No specific species reported	Unknown	Tooth and tongue discoloration
**Zinc**	Toothpastes Mouthwashes	√	√	√	Effective against *Streptococcus mutans, Streptococcus aureus, Streptococcus salivarius, Streptococcus sobrinus, Lactobacillus casei, Porphyromonas gingivalis, Prevotella intermedia, Fusobactereum nucleatum, Treponema denticola, Tanerella forsythia*	Bacteriostatic and bactericidal	Neurological effects like numbness or tingling sensation and nerve damage
**Licorice**	Candies Lollipops Capsules Tablets Liquid extracts	√	√	√	Effective against *Streptococcus mutans, Aggregatibacter actinomycetemcomitans*, *Porphyromonas gingivalis*	Bacteriostatic	Hypokalemia, hypertension, metabolic alkalosis, edema
**Curcumin**	Toothpastes Mouthwashes Gels Powders Capsules Tablets	√	√	√	NA	NA	Allergic contact dermatitis, contact urticaria

√—Positive effect, ×—No effect, NA—Not Available.
